# Sex Differences and Menstrual Cycle Dependent Changes in Cognitive Strategies during Spatial Navigation and Verbal Fluency

**DOI:** 10.3389/fpsyg.2017.00381

**Published:** 2017-03-17

**Authors:** Andrea Scheuringer, Belinda Pletzer

**Affiliations:** ^1^Department of Psychology, University of Salzburg,Salzburg, Austria; ^2^Centre for Cognitive Neuroscience, University of Salzburg,Salzburg, Austria

**Keywords:** sex differences, spatial navigation, verbal fluency, menstrual cycle, cognitive strategies, sex hormones

## Abstract

Men typically outperform women in spatial navigation tasks, while the advantage of women in verbal fluency is more controversial. Sex differences in cognitive abilities have been related to sex-specific cognitive strategies on the one hand and sex hormone influences on the other hand. However, sex hormone and menstrual cycle influences on cognitive strategies have not been previously investigated. In the present study we assessed cognitive strategy use during spatial navigation and verbal fluency in 51 men and 49 women. In order to evaluate sex hormone influences, all participants completed two test sessions, which were time-locked to the early follicular (low estradiol and progesterone) and mid-luteal cycle phase (high estradiol and progesterone) in women. As hypothesized, men outperformed women in navigation, whereas women outperformed men in phonemic verbal fluency. Furthermore, women switched more often between categories in the phonemic fluency condition, compared to men, indicating sex-specific strategy use. Sex differences in strategy use during navigation did, however, not follow the expected pattern. Menstrual cycle phase, however, did modulate strategy use during navigation as expected, with improved performance with the landmark strategy in the luteal, compared to the follicular phase. No menstrual cycle effects were observed on clustering or switching during verbal fluency. This suggests a modulation of cognitive strategy use during spatial navigation, but not during verbal fluency, by relative hormone increases during the luteal phase of the menstrual cycle.

## Introduction

Sex differences in cognitive abilities have been reported most consistently for spatial abilities, with men usually outperforming women (Linn and Peterson, 1985; Voyer et al., 1995; Weiss et al., 2003; Andreano and Cahill, 2009), while women’s superiority in verbal abilities is more controversial (Maccoby and Jacklin, 1974; Hyde and Linn, 1988; Kimura, 2002; Andreano and Cahill, 2009). In both domains, sex differences depend on the type of task and have been related to sex-dependent strategy use on the one hand and sex hormone levels on the other hand. However, it has hardly been previously investigated how sex hormones affect the use of cognitive strategies in spatial and verbal abilities. Since sex hormones have been found to modulate the use of different strategies in other cognitive tasks (Nielsen et al., 2011; Pletzer et al., 2013; Pletzer et al., 2014), this is the focus of the present study.

In the spatial domain, a robust male advantage is evoked especially in mental rotation and navigation tasks (Linn and Peterson, 1985; Voyer et al., 1995; Collins and Kimura, 1997; Astur et al., 1998; Dabbs et al., 1998; Silverman et al., 1999; Saucier et al., 2002; Weiss et al., 2003; Peters, 2005; Mueller et al., 2008a; Puts et al., 2010; Andersen et al., 2012). Among these tasks, sex-dependent strategies are most apparent in spatial navigation, where participants have to find a target location (Gerber and Kwan, 1994). Therefore, a spatial navigation task was selected for the present study. Evidence for sex-dependent strategy use during spatial navigation comes from participants’ self-reports (Lawton, 1994, 1996; Dabbs et al., 1998), recall of map characteristics (Galea and Kimura, 1993), modulation of performance by landmark availability in the environment (Astur et al., 1998; Sandstrom et al., 1998; Andersen et al., 2012), from modulation of performance by the use of different instructions favoring either an allocentric/Euclidian or egocentric/landmark-based strategy (Saucier et al., 2002), as well as from eye-tracking studies investigating differences in duration and patterns of gazing behavior in finding a target (Mueller et al., 2008a; Andersen et al., 2012). These studies suggest that during spatial navigation men take a more allocentric perspective and use a more Euclidian solution strategy, whereas women, take a more egocentric perspective and rely more on a landmark-based strategy (e.g., Ward et al., 1986; Galea and Kimura, 1993; Lawton, 1994; Dabbs et al., 1998; Saucier et al., 2002). Allocentric perspective-taking is characterized by focusing on cardinal directions, like north, east, south, and west, whereas in the egocentric perspective participants rely on personal directions like right or left instead of focusing on absolute or stable cues (Lawton, 1994). In the landmark-based strategy, participants preferentially rely on details, namely concrete landmarks, whereas in the Euclidian strategy, a focus on less detailed and more global landmarks in the environment is described (Lawton, 1994). It is assumed that the landmark-based strategy is more rigid and sequential, whereas the Euclidian strategy should be more flexible, which may therefore lead to an advantage in performance in men (Saucier et al., 2002). For instance, when a wrong turn has been made, people become less disoriented with the Euclidian strategy and its use further leads to an increased possibility to choose between two or more optional routes (Lawton, 1994, 1996).

Several investigations also explain the male advantage in spatial tasks via their high levels of testosterone, since testosterone has repeatedly been related to improved spatial performance (Gordon and Lee, 1986; Silverman et al., 1999; Hausmann et al., 2000, 2009; Aleman et al., 2004; Hooven et al., 2004; Discroll et al., 2005; Burkitt et al., 2007; Yang et al., 2007; Mueller et al., 2008b). Note, however, that some other investigations could not demonstrate any association between testosterone and spatial abilities (McKeever et al., 1987; Puts et al., 2010). They observed a pattern, which is reverse to the intuitive one described above or even speak in favor of a non-linear relationship between testosterone and spatial performance (Shute et al., 1983; Moffat and Hampson, 1996). The role of female sex hormones estradiol and progesterone for spatial abilities is less clear. However, some studies demonstrate that estradiol and progesterone relate to impaired spatial abilities (Hausmann et al., 2000). This is in line with menstrual cycle studies demonstrating improved performance in spatial tasks during the follicular phase, in which estradiol and progesterone levels are low, compared to their luteal phase, in which female sex hormone levels are high (Hausmann et al., 2000; McCormick and Teillon, 2001). However, sex hormone influences on the use of allocentric vs. egocentric perspectives on the one hand and Euclidian vs. landmark-based strategies on the other hand have not been previously investigated. There is only one recent study that has addressed menstrual cycle dependent changes in navigation strategies. Although they define their strategies differently, the strategy enhanced during the luteal cycle phase is characterized by an increased use of landmark information (Hussain et al., 2016).

In the verbal domain, results concerning sex differences are inconsistent when considering large meta-analytic reviews (Maccoby and Jacklin, 1974; Hyde and Linn, 1988; Kimura, 2002; Andreano and Cahill, 2009). One reason for controversial results in verbal abilities may be that large meta-analyses do not differentiate between tasks which require different abilities, and may thus underestimate sex differences in specific tasks (Linn and Peterson, 1985; Voyer et al., 1995; Andreano and Cahill, 2009). A female advantage has most consistently been observed for verbal recall and verbal fluency (Kimura, 2002; Andreano and Cahill, 2009). Among these tasks, sex-dependent strategies have been reported for the verbal fluency task, which was thus selected for the present study. Concerning verbal fluency, larger sex differences, favoring women, have been found for phonemic fluency, which requires participants to produce as many words as possible beginning with a predefined letter (Hyde and Linn, 1988; Capitani et al., 1998; Herlitz et al., 1999; Weiss et al., 2003, 2006; Burton et al., 2005; Halari et al., 2006; Wallentin, 2009; Fillipetti and Allegri, 2011). Sex differences in semantic fluency, in which participants have to generate words belonging to a given category, are usually weaker (Capitani et al., 1999, 2005; Fillipetti and Allegri, 2011; Munro et al., 2012).

Some investigations, however, fail to find a female superiority in verbal fluency at all (Tombaugh et al., 1999; Troyer, 2000; Lewin et al., 2001). Inconsistencies may possibly attributable to different age ranges and education levels of participants in different studies, since age and education has been shown to affect verbal fluency performance (Troyer et al., 1997; Tombaugh et al., 1999; Troyer, 2000; Brucki and Rocha, 2004; Lanting et al., 2009; Sauzéon et al., 2010).

Concerning sex-dependent strategy use during verbal fluency tasks, there is evidence that women produce more clusters and switch more often between categories than men, whereas men generate broader clusters and produce less switches, respectively (Weiss et al., 2006; Lanting et al., 2009). Clustering is described as the consecutive generation of words belonging to the same subcategory (e.g., naming different pets in the category animals) and is defined as relative automatic process involving categorization in semantic fluency and phonemic analysis in phonemic fluency (Troyer et al., 1997). Switching, on the other hand refers to the consecutive generation of words belonging to different sub-categories, i.e., switching to another sub-category when no more words come to mind. Thus, switching requires cognitive flexibility in shifting and is specified as an effortful process (Troyer et al., 1997). Switching, compared to clustering has been suggested to be the more successful strategy for overall verbal fluency performance (Koren et al., 2005; Weiss et al., 2006).

However, not all studies investigating sex differences in verbal fluency strategies were able to detect sex differences (Troyer et al., 1997; Brucki and Rocha, 2004). This may again be attributable to differences in age and education between studies, since both have also been demonstrated to affect switching and clustering, at least to a small degree (Troyer et al., 1997; Troyer, 2000; Brucki and Rocha, 2004; Sauzéon et al., 2010). However, it seems that differences in strategies are stable over a wide age-range (Lanting et al., 2009).

Concerning sex hormone influences on verbal fluency, testosterone tends to have a blocking effect on verbal abilities in general (Van Goozen et al., 1995; Wolf et al., 2000), and verbal fluency in particular, especially in women (Wolf and Kirschbaum, 2002; Thilers et al., 2006). Regarding the female sex hormones estradiol and progesterone, some studies report a positive relationship to verbal fluency performance (Maki et al., 2002). This is consistent with menstrual cycle studies demonstrating an improvement of verbal abilities during the high-hormonal luteal phase compared to the follicular phase (Hampson, 1990a,b; Drake et al., 2000; Rosenberg and Park, 2002). However, menstrual cycle and sex-hormone influences on clustering vs. switching during verbal fluency, have not been previously investigated.

In summary, sex hormone or menstrual cycle influences on strategy use during spatial navigation or verbal fluency have not been previously investigated.

Therefore, in the current experiment we investigate, whether the application of different strategies in navigation and verbal fluency is dependent on female cycle phases and sex hormone levels. We administered a 2-D map-based navigation task and a phonemic and semantic verbal fluency task to a sample of young men and women at two time-points, which were phase-locked to the menstrual cycle in women. Different instructions modulated strategies in both tasks. We expect to replicate the previously demonstrated male advantage in wayfinding and a female superiority in the overall number of words generated, as well as sex-dependent strategies in the navigation task and sex differences in clustering and switching strategies during verbal fluency. We mainly focused on hormone-dependent hypotheses, expecting associations between sex hormones, and thus, menstrual cycle phases and cognitive strategies. Assuming that navigation performance is related to an allocentric perspective and Euclidian strategy use on the one hand, as well as high testosterone levels and low levels of female sex hormones on the other hand, we hypothesize that women perform better with the egocentric and the landmark-based strategy during the luteal as opposed to the follicular cycle phase. Also, assuming that verbal fluency performance is related to switching on the one hand and high levels of female sex hormones, but low levels of testosterone on the other hand, we hypothesize that women produce larger clusters and less switches in the verbal fluency task during the follicular compared to the luteal cycle phase. Concomitant with this hypothesis is the assumption of a positive association between testosterone and allocentric/Euclidian strategies during navigation, as well as clustering during verbal fluency, as well as a positive relationship between estradiol and progesterone and egocentric/landmark based strategies during navigation, as well as switching during verbal fluency, respectively.

## Materials and Methods

### Participants

One-hundred German-speaking participants, 51 men (*M*_age_ = 23.65, *SD* = 4.06) and 49 women (*M*_age_ = 22.57; *SD* = 3.19), completed the present study. Age of all participants ranged between 18 and 36 and did not differ significantly between men and women [*t*_(98)_ = −1.47, *p* = 0.15]. Participants were students of the University of Salzburg, who received course credits for their participation. They had all completed their A-Levels and were thus at a comparably high level of education.

Only participants who reported to be right-handers and had no history of neurological, psychological or endocrinological disorders were included in the study. Women were only allowed to participate, if they reported no use of hormonal contraceptives and a regular menstrual cycle of a duration between 21 and 36 days. The mean cycle length of women in the present study was 29.24 days (*SD* = 2.64). All participants were tested twice. While men participated within an interval of around 2 weeks, women participated in two different cycle phases, i.e., the low-hormonal early follicular phase (days 1–6 of their menstrual cycle) and the high-hormonal mid-luteal phase (3–10 days after ovulation). Ovulation was calculated based on participants’ self-reports and was confirmed by commercial ovulation tests and by hormone levels analyzed in saliva after testing. Twenty-three women were in their follicular cycle phase during the first session and were tested in their luteal cycle phase in the second session. 26 women were tested in their luteal cycle phase in the first session and in their follicular phase in the second session, respectively.

### Ethics Statement

All participants gave their written consent to participate in the study and all methods went conform to the Code of Ethics of the World Medical Association (Declaration of Helsinki).

The institutional guidelines of the University of Salzburg (Statutes of the University of Salzburg^1^) state in §163 (1) that ethical approval is necessary for research on human subjects if it affects the physical or psychological integrity, the right for privacy or other important rights or interests of the subjects or their dependents. In §163 (2) it is stated that it is the responsibility of the PI to decide, whether (1) applies to a study or not. Therefore, we did not seek ethical approval for this study. Since it was non-invasive and performed on healthy adult volunteers, who gave their informed consent to participate, (1) did not apply. Data was processed in anonymized/de-identified form. Upon arrival at the lab, participants were assigned a subject ID (v001, v002, etc.), which was used throughout the study.

### Navigation Task

**FIGURE 1:**
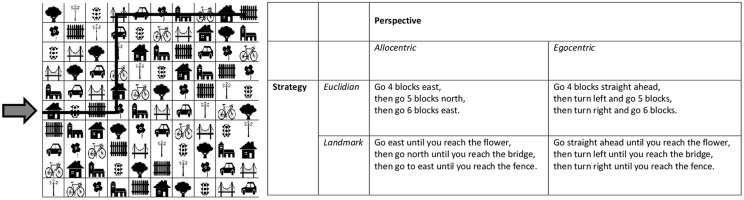
**An example of the 2-D matrix navigation task is given.** The arrow highlights the beginning of the trial. The black line through the matrix marks the path which is described in the matrix on the right in four different possible ways. Different descriptions represent combinations of one type of perspective and one type of strategy description.

To evaluate spatial navigation abilities, as well as their preferred perspective and choice of strategy, participants completed a computerized 2D-matrix navigation task. The task was adapted from an earlier paper-pencil version by Saucier et al. (2002). As in Saucier et al. (2002), each item was comprised by a 10 × 10 matrix containing 10 different symbols, which were repeated once in each row and column. The order of the symbols varied between trials. Symbols or their names, respectively, were selected from very common and simple German words, which are included in the basic vocabulary, learnt in the 1st years of elementary school (Grasmück et al., 2014) and included a tree, flower, house, church, bridge, lantern, fence, car, bicycle, and traffic light (for an example, see **Figure 1**).

For each item participants received a short written instruction of three lines, which was presented for 15 s before the matrix appeared. As in Saucier et al. (2002), each instruction described a path on the matrix. The beginning of the path was marked by a red arrow. Participants were supposed to move the arrow along the path using the arrow keys which allowed them to move from one cell of the matrix to another. As in Saucier et al. (2002), each path extended over 15 fields and included 2 turns. The beginning of the path was either on the left or the right side, or the top or the bottom of the matrix. The trial ended by pressing the enter key.

Instructions were formulated as in Saucier et al. (2002) to compare two possible perspectives (i.e., Allo- vs. egocentric) and two possible strategies (i.e., Euclidian vs. landmark-based). However, while in the original version by Saucier et al. (2002), the allocentric perspective was always combined with the Euclidian strategy and the egocentric perspective with the landmark-based strategy, perspective and strategy were manipulated in a 2 × 2 design in the current study. Example instructions are included in **Figure 1**. For every type of instruction, 4 trials were used, resulting in a total of 16 different trials. All participants received all items and the order of trials was randomized across participants.

Response times and accuracy were recorded for each item. Response times were analyzed only for correctly solved items. Participants needed about 15 min to complete this task.

### Verbal Fluency Task

To evaluate verbal abilities, as well as clustering and switching strategies, participants completed a paper-pencil version of the verbal fluency task. For participants, aged 21–29 of all education levels, an average production of 8.33 words per minute is suggested for a written version of the phonemic fluency task (Horn, 1983). Both semantic and phonemic fluency were assessed and counter-balanced across participants. Each condition included different trials with three different instructions. Within each instruction, 51 participants received the phonemic fluency condition first, and 49 participants received the semantic fluency condition first. In each condition, participants were given a time limit of 1 min. In the phonemic fluency condition, participants are asked to write down as many words as possible on a piece of paper, beginning with a predefined letter. In the semantic condition, respectively, participants were asked to name as many words as possible belonging to a given semantic category (e.g., *animals*). To manipulate strategy use, different instructions were used. While the neutral instruction simply requested participants to name as many words as possible, the clustering instruction required them to write down consecutive words, which should be as similar as possible in the phonemic condition (i.e., consecutive words should have as many identical letters as possible, should only differ in one vocal, or should rhyme) and should belong to the same sub-category in the semantic condition, respectively (e.g., for the main category animals, words produced belong to subcategories like *fish* and *birds*). In the switching instruction, consecutive words should be as dissimilar as possible in the phonemic condition (i.e., successive words should consist of as many different letters as possible, except the initial letter, or they should not rhyme), or belong to different sub-categories in the semantic condition. Letters, used in the present phonemic condition were the consonants *P*, *N*, *M*, *S*, *H*, *L*, *F*, *T*, *D*, *W*, *K*, and *R*. For the semantic fluency condition, we used the following categories: *animals*, *fruits*, *beverages*, *food*, *furniture*, *clothing*, *jobs*, *cities*, *countries*, *toys*, *sports*, and *means of transportation*. For each participant, two different letters and two different categories were randomly assigned to each instruction and test session. Thus, altogether, in one session participants received a booklet with six different initial letters and six different categories. For both tasks, we formed letter- or category pairs, respectively, such that the frequency of German words beginning with these letters or belonging to this category, approximately matched across instructions and test sessions (*P*/*N*, *M*/*S*, *H*/*L, F*/*T*, *D*/*W*, *K*/*R and jobs*/*countries*, *furniture*/*animals*, *food*/*toys, beverages*/*cities*, *clothes*/*fruits*, *means of transportation*/*sports).*

For completing both conditions participants needed about 20 min. Performance on this task was measured by computing the mean number of words produced for each verbal fluency condition. Additionally, the mean cluster size, and the mean number of switches was measured for every task and instruction, assessing participant’s strategies used in the test. Clusters and switches were counted using the rules suggested by Troyer et al. (1997). Homophones were furthermore not considered when counting words due to the written test format.

### Statistical Analyses

Statistical analyses were carried out in R 3.3.1. Linear mixed effects models were utilized using the *lme* function of the *lme4* package. Dependent variables were reaction time (RT) in milliseconds and accuracy (percentage of correct responses) for the navigation task, and number of words, cluster size and number of switches in the verbal fluency task, respectively.

The advantages of the lme approach for the current research questions are threefold: (i) It is possible to control for learning effects by including session as a fixed effect even in the menstrual cycle models, where these within-subjects effects are crossed. (ii) Categorical effects with more than one category are automatically dummy coded, thus comparing each category to a reference category, which renders *post hoc* comparisons unnecessary. (iii) Sex hormone influences can be evaluated across all time-points and conditions at once and are compared between men and women via interaction effects in the model. Thus separate correlation analyses and the *post hoc* comparison of correlation coefficients are not necessary. To make use of these advantages, we did test the following models:

To control for repeated measurement in the linear mixed effect models, ‘*participant number*’ was treated as random factor. In all tasks, the factor ‘*session*’ was included as a fixed factor to the model in order to control for possible memory or learning effects. For each task, sex differences in strategy use were evaluated, by models including the interactive effects of ‘*instruction*’ and ‘*sex.*’ Cycle-dependent changes were evaluated in the female sub-sample by models including the interactive effects of ’*instruction*’ and ‘*cycle.*’ Specific models for each task are described in the section “Results.” To evaluate the effect of sex hormones on the dependent variables, ‘*progesterone*,’ ‘*estradiol*,’ and ‘*testosterone*,’ were separately added as additional fixed effects to these models.

Non-significant interactions were backward eliminated like implemented in the step function of the *lmerTest* package to create minimum models including only the factors and interactions which are relevant to explain the dependent variables. Results of these minimum models are reported.

In all models, both, the dependent and continuous independent variables were z-standardized using the *scale* function. Therefore, the coefficients b of fixed effects in the models represent a standardized effect size based on standard deviations, similar to Cohen’s *d*.

### Hormone Levels

Sex hormone levels were assessed from saliva samples. Three saliva samples were taken from each participant during each session. The first saliva sample was taken after filling out the informed consent, when participants had time to adjust to the lab environment. The second saliva sample was taken between the tasks, whereas the last one was taken at the end of the study, before participants received debriefing, if requested. Saliva samples were stored at −20°C and centrifuged at 3000 rpm for 20 min prior to hormone assessment. Testosterone, 17β-estradiol and progesterone were assessed for each sample using ELISA kits from DeMediTec. Hormone levels were averaged over the three samples obtained during one session, in order to control for diurnal fluctuations in hormone levels and ensure reliability of hormone assessment. Five women had to be excluded from analyses because of inconsistencies between the self-reported cycle phases and the analyzed hormone values. Additional 2 men were excluded from further statistical analyses because testosterone levels varied highly between the two test sessions, suggesting external factors influencing testosterone levels, like stress. Thus, all analyses were performed on 49 men and 44 women.

Additionally, 2 estradiol levels, 7 progesterone levels and 3 testosterone levels were missing as they could not be analyzed due to insufficient sample volume.

To evaluate sex differences regarding hormone levels, separate linear mixed effects models (lmes) for each hormone including ‘*participant number*’ as a random factor and ‘*sex*’ and ‘*session*’ as fixed factors were run using the *lme* function of the *lme4* package in R 3.3.1. (e.g., Testosterone ∼ 1| PNr + session^∗^sex). As expected, men had higher testosterone levels [*b* = 1.05, *SE*_b_ = 0.17, *t*_(90)_ = 6.01, *p* < 0.001; men: *M* = 173.74, *SE* = 9.22; women: *M* = 71.96, *SE* = 9.79] and lower estradiol [*b* = −0.68, *SE*_b_ = 0.19, *t*_(90)_ = −3.47, *p* < 0.001; men: *M* = 2.86, *SE* = 0.20, women: *M* = 3.97, *SE* = 0.21] and progesterone levels [*b* = −0.72, *SE*_b_ = 0.21, *t*_(89)_ = −3.63, *p* < 0.001; men: *M* = 76.15, *SE* = 2963 women: *M* = 237.0780, *SE* = 30.80] compared to women with no differences between test sessions (all |*b|* < 0.18, all *SE*_b_ > 0.11, all |*t|* < 1.24, all *p* > 0.21).

To evaluate cycle differences, separate lmes including ‘*cycle phase*’ as a fixed factor were run for each hormone in the female sub-sample (e.g., Testosterone ∼ 1| PNr + cycle). Testosterone differed slightly between cycle phases [*b* = 0.30, *SE*_b_ = 0.15, *t*_(42)_ = 2.04, *p* = 0.047; follicular phase: *M* = 65.62, *SE* = 6.39; luteal phase: *M* = 78.25, *SE* = 6.39], while estradiol (follicular phase: *M* = 3.61, *SE* = 0.28; luteal phase: *M* = 4.35, *SE* = 0.28) and progesterone (follicular phase: *M* = 128.07, *SE* = 46.74; luteal phase: *M* = 345.79, *SE* = 46.74) were significantly higher in the luteal, compared to the follicular cycle phase (both *b* > 0.38, both *SE*_b_ < 0.16, both *t* > 0.2.42, both *p* < 0.020).

## Results

### Navigation Task

#### Sex Differences

**FIGURE 2:**
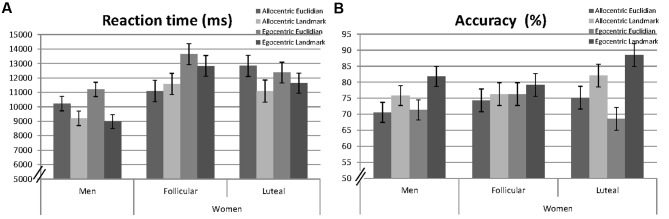
**Mean reaction times (milliseconds, A)** and accuracy (percentage of hits, **B**) to allocentric and egocentric items, when combined with Euclidian or landmark-based descriptions in men, averaged over both sessions and in women, separately for the follicular and the luteal cycle phase. Error bars represent standard errors of the mean (SE).

Linear mixed effects models were fitted for reaction times and accuracy including participant number (PNr) as a random factor and the fixed effects session, as well as the interactive effects of ‘*perspective*,’ ‘*strategy*,’ and ‘*sex*’ (e.g., RT ∼ 1| PNr + session + perspective^∗^strategy^∗^sex). Non-significant interactions were backward eliminated and results of the final models are reported. Specifically, the threefold interaction was always non-significant and thus removed from the model. Mean response times and the mean number of hits for men and women are presented in **Figure 2**.

##### Reaction times

A significant main effect of ‘*session*’ indicated faster reaction times during the second, compared to the first session [*b* = −0.22, *SE*_b_ = 0.04, *t*_(2155)_ = −5.82, *p* < 0.001]. A significant main effect of ‘*sex*’ [*b* = −0.36, *SE*_b_ = 0.09, *t*_(91)_ = −3.91, *p* < 0.001] indicated that men were significantly faster in solving navigation items, compared to women. Significant main effects of ‘*perspective*’ [*b* = −0.10, *SE*_b_ = 0.04, *t*_(2155)_ = 2.75, *p* = 0.006] and ‘*strategy*’ [*b* = −0.19, *SE*_b_ = 0.04, *t*_(2155)_ = −5.06, *p* < 0.001] indicated that reactions were faster during the allocentric, compared to the egocentric perspective and with the landmark strategy, compared to the Euclidian strategy. All interactions were non-significant and thus removed from the model.

##### Accuracy

A significant main effect of ‘*session*’ indicated higher accuracy during the second session [*b* = 0.21, *SE*_b_ = 0.03, *t*_(2861)_ = 6.21, *p* < 0.001]. The main effect of ‘*sex*’ was non-significant and therefore removed from the model. A significant main effect of ‘*strategy*’ [*b* = 0.11, *SE*_b_ = 0.05, *t*_(2861)_ = 2.41, *p* = 0.016] indicated higher accuracy with the landmark-based strategy, compared to the Euclidian strategy. The main effect of ‘*perspective*’ was, however, non-significant [*b* = −0.02, *SE*_b_ = 0.05, *t*_(2861)_ = −0.35, *p* = 0.728], but remained in the model due to a significant ‘*perspective*’^∗^’*strategy*’ interaction [*b* = 0.14, *SE*_b_ = 0.07, *t*_(2861)_ = 2.11, *p* = 0.035]. The ‘*strategy*’ effect was larger with the egocentric perspective, compared to the allocentric perspective, i.e., accuracy was particularly high with the landmark strategy under the egocentric perspective. Neither ‘*perspective*,’ nor ‘*strategy*’ interacted significantly with ‘*sex.*’ Therefore, these interactions were removed from the model.

#### Menstrual Cycle Effects

For female participants, further linear mixed effects models were fitted for reaction times and accuracy including ‘*participant number*’ (PNr) as a random factor and the fixed effects ‘*session*,’ as well as ‘*perspective*,’ ‘*strategy*,’ and ‘*cycle*’ (e.g., RT ∼ 1| PNr + session + perspective^∗^strategy^∗^cycle). Non-significant interactions were backward eliminated and results of the final models are reported. Specifically the threefold interaction was always non-significant and thus removed from the model.

##### Reaction times

Significant main effects of ‘*session*’ [*b* = −0.21, *SE*_b_ = 0.06, *t*_(1041)_ = −3.79, *p* < 0.001] and ‘*perspective*’ [*b* = 0.25, *SE*_b_ = 0.08, *t*_(1041)_ = 3.22, *p* = 0.001] were confirmed. The main effects of ‘*strategy*’ and ‘*cycle phase*’ [*b* = 0.08, *SE*_b_ = 0.08, *t*_(1041)_ = 0.99, *p* = 0.321] were, however, non-significant. ‘*Strategy*’ did not interact with ‘*perspective*’ or ‘*cycle-phase*’ and was thus removed from the model. However, a significant interaction between ‘*cycle phase*’ and ‘*perspective*’ was observed [*b* = −0.25, *SE*_b_ = 0.11, *t*_(1041)_ = −2.25, *p* = 0.025]. During the luteal cycle phase reaction times for the allocentric instruction increased, while reaction times for the egocentric instruction decreased compared to the follicular cycle phase (compare **Figure 2A**).

##### Accuracy

A significant main effect of ‘*session*’ [*b* = 0.21, *SE*_b_ = 0.05, *t*_(1356)_ = 4.36, *p* < 0.001] was confirmed. The main effects of ‘*strategy*,’ ‘*perspective*,’ and ‘*cycle*’ were non-significant. ‘*Perspective*’ did not interact with ‘*cycle*’ or ‘*strategy*’ and was thus removed from the model. The main effects of ‘*strategy*’ [*b* = 0.06, *SE*_b_ = 0.07, *t*_(1356)_ = 0.81, *p* = 0.420] and ‘*cycle*’ [*b* = −0.08, *SE*_b_ = 0.07, *t*_(1356)_ = −1.16, *p* = 0.245] were, however, qualified by a significant strategy^∗^cycle interaction [*b* = 0.26, *SE*_b_ = 0.10, *t*_(1356)_ = 2.64, *p* = 0.008] and thus remained in the model. During the luteal cycle phase, accuracy for the Euclidian strategy decreased, while accuracy for the landmark strategy increased compared to the follicular cycle phase (compare **Figure 2B**).

#### Modulation by Sex Hormones

In order to evaluate, whether sex hormones impacted overall performance or strategy use during navigation, ‘*progesterone*,’ ‘*estradiol*,’ and ‘*testosterone*’ levels were separately added as predictors to the models described above (e.g., RT ∼ 1| PNr + session + perspective^∗^strategy^∗^sex^∗^testosterone). Since separate models were run for each of the three hormones, the significance level was adjusted to *p* = 0.016. Non-significant interactions and main effects were backward eliminated. Neither ‘*testosterone*’ nor ‘*estradiol*’ or ‘*progesterone*’ had a significant effect on RT.

For accuracy, a significant interaction between ‘*progesterone*’ and ‘*perspective*’ was found [*b* = −0.14, *SE*_b_ = 0.03, *t*_(2703)_ = −4.23, *p* < 0.001]. The higher the progesterone levels, the larger was the perspective effect, i.e., the higher was the accuracy for the egocentric perspective and the lower was the accuracy for the allocentric perspective. ‘*Testosterone*’ and ‘*estradiol*’ did not affect navigation accuracy.

### Verbal Fluency Task

#### Sex Differences

**FIGURE 3:**
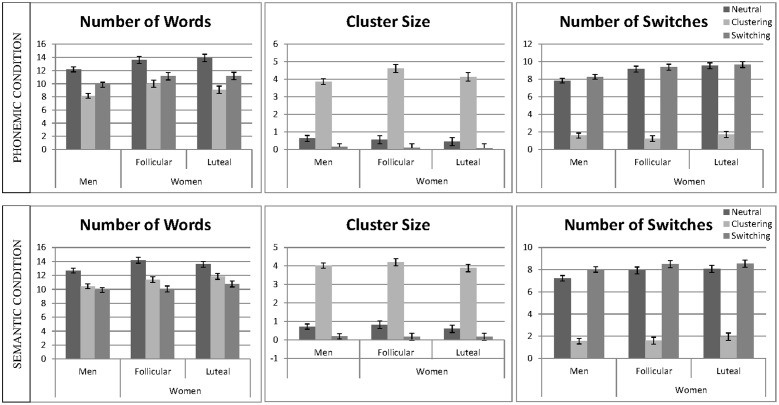
**Verbal fluency performance and strategy use.** The mean number of words, cluster size and number of switches are displayed separately for the phonemic condition **(top)** and semantic condition **(bottom)**. Means are given for men, averaged over both sessions and women, separately for the follicular and the luteal cycle phase, for the neutral, the clustering and the switching condition. Error bars represent standard errors of the mean (SE).

Linear mixed effect models were calculated for the number of words, average cluster size, and number of switches, including ‘*participant number*’ as a random factor and ‘*session*,’ as well as the interactive effects of ‘*instruction*’ and ‘*sex*’ as fixed effects, separately for the phonemic and the semantic condition (e.g., words ∼ 1| PNr + session + instruction^∗^sex). Non-significant interactions were backward eliminated and results of the final models are reported. The mean number of words, the cluster size, and the mean number of switches for the phonemic and the semantic task are displayed in **Figure 3**.

##### Number of words

In the semantic condition, the main effect of ‘*session*’ was non-significant and thus removed from the model. In the phonemic condition, the main effect of ‘*session*’ was significant, i.e., participants produced significantly more words during the second as opposed to the first session [*b* = 0.18, *SE*_b_ = 0.06, *t*_(438)_ = 2.89, *p* = 0.004]. In both conditions, the main effects of *‘instruction’* were significant, i.e., participants produced significantly more words during the neutral instruction as opposed to the clustering instruction [phonemic: *b* = −1.12, *SE*_b_ = 0.08, *t*_(438)_ = −14.49, *p* < 0.001; semantic: *b* = −0.72, *SE*_b_ = 0.08, *t*_(428)_ = −9.03, *p* < 0.001] and switching instruction [phonemic: *b* = −0.67, *SE*_b_ = 0.08, *t*_(438)_ = −8.81, *p* < 0.001; semantic: *b* = −1.00, *SE*_b_ = 0.08, *t*_(428)_ = −12.65, *p* < 0.001]. In both conditions, the main effect of sex was significant, i.e., women produced significantly more words than men [phonemic: *b* = −0.39, *SE*_b_ = 0.12, *t*_(90)_ = −3.33, *p* = 0.001; semantic: *b* = −0.31, *SE*_b_ = 0.12, *t*_(90)_ = −2.48, *p* = 0.015]. In both, the phonemic and the semantic condition, all interactions were non-significant and thus removed from the model.

##### Cluster size

In both conditions, the main effect of ‘*session*’ was non-significant and thus removed from the model. In both conditions, the main effect of *‘instruction’* was significant, i.e., participants produced significantly larger clusters during the clustering instruction, as opposed to the neutral instruction [phonemic: *b* = 1.49, *SE*_b_ = 0.07, *t*_(427)_ = 22.01, *p* < 0.001; semantic: *b* = 1.54, *SE*_b_ = 0.07, *t*_(428)_ = 23.70, *p* < 0.001] and significantly smaller clusters during the switching instruction compared to the neutral instruction [phonemic: *b* = −0.19, *SE*_b_ = 0.07, *t*_(427)_ = −2.74, *p* = 0.006; semantic: *b* = −0.25, *SE*_b_ = 0.06, *t*_(428)_ = −3.82, *p* < 0.001]. In both conditions the main effect of ‘*sex*’ was non-significant, i.e., cluster size did not differ significantly between men and women. The effect was removed from the model, since in both condition, the interactions were furthermore non-significant.

##### Number of switches

In the semantic condition, the main effect of ‘*session*’ was non-significant and thus removed from the model. In the phonemic condition, the main effect of ‘session’ was significant, indicating a larger number of switches during the second compared to the first session [*b* = 0.15, *SE*_b_ = 0.04, *t*_(434)_ = 3.71, *p* < 0.001]. In both conditions, the main effect of the switching ‘*instruction*’ was significant, i.e., participants produced a smaller number of switches in the clustering instruction, as opposed to the neutral instructions [phonemic: *b* = 0.04, *SE*_b_ = 0.07, *t*_(434)_ = 0.58, *p* = 0.563; semantic: *b* = −1.64, *SE*_b_ = 0.06, *t*_(421)_ = −28.57, *p* < 0.001], but only in the semantic condition did they produce a larger number of switches during the switching instruction, as opposed to the neutral instruction [phonemic: *b* = −1.91, *SE*_b_ = 0.07, *t*_(434)_ = −25.85, *p* < 0.001; semantic: *b* = 0.18, *SE*_b_ = 0.06, *t*_(421)_ = 3.19, *p* = 0.002]. The main effect of ‘*sex*’ was significant in both conditions [phonemic: *b* = −0.37, *SE*_b_ = 0.09, *t*_(90)_ = −3.84, *p* < 0.001, semantic: *b* = −0.14, *SE*_b_ = 0.07, *t*_(90)_ = −2.02, *p* = 0.047], indicating a higher number of switches in women compared to men. In the semantic conditions the interaction between ‘*sex*’ and ‘*instruction*’ was non-significant and thus removed from the model. In the phonemic condition, the interaction between ‘*sex*’ and ‘*instruction*’ was significant for the comparison of clustering and neutral instruction [*b* = 0.40, *SE*_b_ = 0.10, *t*_(434)_ = 3.90, *p* < 0.001], but not for the comparison of switching and neutral instruction [*b* = 0.06, *SE*_b_ = 0.10, *t*_(434)_ = 0.65, *p* = 0.515], indicating that women reduced their switches more strongly during the clustering condition as opposed to the neutral condition than men.

#### Menstrual Cycle Effects

In the female sample, further linear mixed effects models were fitted for the number of words, the cluster size and the number of switches including ‘*participant number*’ (PNr) as a random factor and the fixed effects ‘*session*,’ as well as the interactive effects of ‘*instruction*’ and ‘*cycle*’ (e.g., RT ∼ 1| PNr + session + instruction^∗^cycle), separately for the phonemic and the semantic condition. Non-significant interactions were backward eliminated. Main effects of ‘*session*’ and ‘*instruction*’ were confirmed as in the analyses of sex differences (compare Sex Differences). No significant results were observed for the main effect of ‘*cycle*’ and the ‘*cycle*’^∗^‘*instruction*’ interaction, neither for the number of words, nor for cluster size, nor for the number of switches. ‘*Cycle phase*’ was therefore removed from all models.

#### Modulation by Sex Hormones

In order to evaluate the impact of sex hormone levels on verbal fluency performance and strategies, ‘*progesterone*,’ ‘*estradiol*,’ and ‘*testosterone*’ levels were separately added as predictors to the models described above. (e.g., RT ∼ 1| PNr + session + instruction^∗^sex^∗^testosterone). Since separate models were run for each of the three hormones in two task conditions, the significance level was adjusted to *p* = 0.008. Non-significant interactions and main-effects were backward eliminated. Under the corrected threshold, neither testosterone, nor progesterone or estradiol had an influence on the total number of words produced, the cluster size or the number of switches in any task condition.

## Discussion

The main objective of this study was to investigate sex differences and sex hormone influences on overall performance and especially on the use of strategies in a spatial navigation task and in a verbal fluency task. We expected to replicate a male advantage in navigation and the use of allocentric perspectives and Euclidian strategies, as well as a female advantage in verbal fluency and preferred switching between categories. We expected female menstrual cycle phases and sex hormone levels to affect performance and strategy use in both tasks.

We were able to confirm a male advantage in mean reaction times of the spatial navigation task and a female advantage in the verbal fluency task.

However, menstrual cycle phase did not affect overall performance in either task. While we confirmed sex differences in switching during verbal fluency, there were no sex differences in perspective and strategy use during navigation. Menstrual cycle phase did modulate the effect of strategy and perspective during navigation, whereas no menstrual cycle effects on clustering and switching during verbal fluency were observed. In the following these results will be discussed in further detail.

### Sex and Menstrual Cycle Differences in Overall Performance

For the navigation task the male advantage was clearly visible, with reference to faster response times, but not with reference to accuracy. Performance differences in reaction times, favoring men have also been described for mental rotation tasks (Maccoby and Jacklin, 1974; Linn and Peterson, 1985) and discussed as reflecting more impulsive decision making in men as opposed to more careful decision making in women. Also, in mental rotation, a male advantage arises especially, when women are pressed for time (Goldstein et al., 1990). Thus, if no time limit is set, such as in the present study, more careful decision making in women leads to comparable accuracy but slower reaction times in comparison to men. Furthermore, it has often been discussed, that 2-D navigation tasks evoke smaller sex differences in accuracy than 3-D navigation tasks (Galea and Kimura, 1993; Saucier et al., 2002; Andreano and Cahill, 2009).

Concerning verbal fluency, the present study revealed that women generated overall more words than men in both fluency conditions. This is in line with the literature, although more studies speak in favor of a female advantage in the phonemic fluency task (Hyde and Linn, 1988; Capitani et al., 1998; Herlitz et al., 1999; Weiss et al., 2003, 2006; Burton et al., 2005; Halari et al., 2006; Wallentin, 2009; Fillipetti and Allegri, 2011), while there are more controversies concerning sex differences in semantic fluency (Capitani et al., 1999, 2005; Tombaugh et al., 1999; Fillipetti and Allegri, 2011; Munro et al., 2012).

Unlike previous studies (Hampson, 1990a,b), we were not able to demonstrate cycle- dependent differences in overall performance for the navigation or the verbal fluency task. However, reports of menstrual cycle changes in spatial and verbal performance have been discussed controversially due to small sample sizes and inconsistencies in the determination of cycle phases (Sundström-Poromaa and Gingnell, 2014). The present study was performed on a sufficiently large sample of young women with strict exclusion criteria regarding the menstrual cycle phases in question. It has also been discussed that menstrual cycle dependent changes in verbal and spatial performance may rather be related to a rise in estradiol during the late follicular phase (Sundström-Poromaa and Gingnell, 2014), which was not investigated in the present study and would be of special interest in future investigations.

### Sex Differences in Cognitive Strategies

In the navigation task a general advantage was observed when applying the landmark-based strategy, compared to the Euclidian strategy and with the allcoentric, compared to the egocentric perspective, for both, men and women. These observations are contrary to our hypotheses, as an advantage for the allocentric perspective, compared to the egocentric perspective, was expected only for men, while for women better performance with the egocentric perspective was expected. Furthermore, an advantage with landmark-based compared to Euclidian strategies was expected only for women, while for men an advantage with Euclidian strategies was expected.

It has been demonstrated that the perceptual salience of different stimulus aspects can alter processing strategies in other tasks (Martin, 1979; Kimchi and Palmer, 1982; Hughes et al., 1990; Kimchi, 1992). As the size of the landmark symbols in the 2D matrix navigation task was quite large, it is possible that the perceptual salience of the landmarks diminished a preference for Euclidian distances in men.

Another problem with the computerized 2D-matrix format, which may have influenced the effect of perspective, is that the egocentric instructions require mental rotation. As the matrix does not rotate if the participant takes a turn, a right turn when facing downward for example requires a left button response. With the allocentric instructions, however, mental rotation is not necessary, since the north of the matrix is always fixed to the top of the computer screen. Thus, sex differences in mental rotation ability may have confounded the sex differences in perspective preference, which is a limitation of the present study.

We were thus not able to replicate findings on sex-typical strategies use and perspective preferences (Galea and Kimura, 1993; Lawton, 1994; Astur et al., 1998; Dabbs et al., 1998; Saucier et al., 2002; Andersen et al., 2012).

With respect to cycle dependent changes, the landmark-based strategy evoked better accuracy in the luteal phase, compared to the follicular phase and the allocentric perspective evoked faster reactions in the follicular phase compared to the luteal phase in women, as expected. These observations are in line with previous studies demonstrating a stronger focus on stimulus-details or local elements of a stimulus in the luteal phase of the menstrual cycle (Nielsen et al., 2011; Pletzer et al., 2014). Landmarks can be argued to represent the details or local elements of the navigation matrix. Furthermore, one recent study was also able to demonstrate an increase in so called spatial strategies, focusing on landmarks during the luteal phase of the menstrual cycle (Hussain et al., 2016).

With the verbal fluency task, we were able to confirm our hypothesis concerning sex differences in strategies, when considering the number of switches. Women switched significantly more often between clusters than men. Analyses of the cluster size, however, did not evoke sex differences, which is contrary to previous results (Weiss et al., 2006). However, there is evidence that clustering and switching are separate mechanism, with switching being more important for overall performance, at least on phonemic fluency (Troyer et al., 1997, 1998; Troyer, 2000; Kavé et al., 2008). This could be confirmed in the present study, when adding the number of switches and cluster size as a fixed effect to analysis of overall verbal fluency performance. The number of switches, but not cluster size, was a significant predictor of the number of words produced. Thus, sex differences in switching may explain sex differences in overall performance in the phonemic task. Like for overall performance, no menstrual cycle dependent changes were observed in clustering or switching in the verbal fluency task.

### Relationship to Sex Hormone Levels

Against our hypothesis, testosterone levels did not affect performance in the navigation or verbal fluency task. These results are contrary to numerous previous findings suggesting a positive effect of testosterone on spatial performance and a negative effect on verbal fluency (Gordon and Lee, 1986; Van Goozen et al., 1995; Silverman et al., 1999; Hausmann et al., 2000, 2009; Aleman et al., 2004; Hooven et al., 2004; Discroll et al., 2005; Thilers et al., 2006; Burkitt et al., 2007; Mueller et al., 2008b). However, the literature regarding sex hormone influences on cognitive performance is by no means consistent and several studies have – like the present study – suggested opposite relationships or even inverted u-shaped relationships (Shute et al., 1983; McKeever et al., 1987; Gouchie and Kimura, 1991; Kampen and Sherwin, 1996; Moffat and Hampson, 1996; Janowsky et al., 1998; Halari et al., 2005; Puts et al., 2010).

However, as hypothesized, we found progesterone to modulate the perspective effect, which is in line with the menstrual cycle dependent changes observed in the present study. The higher the progesterone, as is the case during the luteal cycle phase, the less allocentric and the more egocentric becomes women’s perspective.

Furthermore, neither estradiol nor progesterone affected performance or strategies in the verbal fluency task. This is consistent with the finding of no menstrual cycle influences on verbal fluency performance or strategies and suggests that sex differences in verbal fluency performance are more a result of organizational rather than activational effects of sex hormones. In line with this conclusion, previous studies have demonstrated sex differences in verbal abilities already in children, i.e., before the onset of puberty (Kramer et al., 1997; Bornstein et al., 2004; Logan and Johnston, 2010; compare Andreano and Cahill, 2009 for a review).

#### Limitations

Even though all participants were university students with similar education levels, we did not explicitly control for general intelligence in the present study. However, if results of the present study were due to insufficient matching in general intelligence, we would expect one group to perform better in both tasks. We did, however, observe better performance of men in the navigation task and better performance of women in the verbal fluency task, such that potential differences in general intelligence cannot fully explain these effects.

A potential confound of the egocentric perspective in the navigation task with mental rotation ability has already been discussed as a limitation for the interpretation of sex differences in perspectives during the navigation task.

Another limitation with the navigation task is that participants had to remember the route-descriptions. Individual differences in memory abilities may therefore also have biased possible sex differences.

### Summary

In summary, the present study confirms a robust male advantage, with respect to reaction times in the navigation task, and a robust female advantage, in the phonemic verbal fluency condition. Furthermore, the present study replicates previous reports of sex differences in strategy use in the verbal fluency task, while the interpretation of sex differences in navigation strategies is limited by the current format of the task. These limitations do, however, not affect the interpretation of within-subjects menstrual cycle comparisons in the female sample. An increased preference for the landmark-based strategy was confirmed for the luteal cycle phase, compared to the follicular phase of women. However, menstrual cycle does not affect strategy use during the verbal fluency task.

## Author Contributions

BP designed and made the concept of the study. AS was responsible for data acquisition. Analysis and interpretation of the data were performed by both authors, BP and AS. AS drafted the manuscript, which was critically revised and finally approved by BP. Both authors agree to be accountable for all aspects of the work in ensuring that questions related to the accuracy or integrity of any part of the work are appropriately investigated and resolved.

## Conflict of Interest Statement

The authors declare that the research was conducted in the absence of any commercial or financial relationships that could be construed as a potential conflict of interest.

## Footnotes

1 https://online.uni-salzburg.at/plus_online/wbMitteilungsblaetter.display?pNr=98160
